# Factors Associated with Postoperative Lipiduria and Hypoxemia in Patients Undergoing Surgery for Orthopedic Fractures

**DOI:** 10.3389/fsurg.2022.814229

**Published:** 2022-04-28

**Authors:** Chih-Hui Chen, Yun-Che Wu, Yu-Cheng Li, Feng-An Tsai, Jen-Ying Li, Jun-Sing Wang, Cheng-Hung Lee

**Affiliations:** ^1^ Department of Orthopedics, Taichung Veterans General Hospital, Taichung, Taiwan; ^2^ Department of Medicine, School of Medicine, National Yang Ming Chiao Tung University, Taipei, Taiwan; ^3^ Department of Orthopedic surgery, Changhua Christian Hospital, Changhua, Taiwan; ^4^ Department of Pathology & Laboratory Medicine, Taichung Veterans General Hospital, Taichung, Taiwan; ^5^ Division of Endocrinology and Metabolism, Department of Internal Medicine, Taichung Veterans General Hospital, Taichung, Taiwan; ^6^ Ph.D. Program in Translational Medicine, National Chung Hsing University, Taichung, Taiwan; ^7^ Department of Food Science and Technology, Hung Kuang University, Taichung, Taiwan

**Keywords:** fracture, hypoxemia, lipiduria, orthopedics, surgery

## Abstract

We investigated factors associated with postoperative lipiduria and hypoxemia in patients undergoing surgery for orthopedic fractures. We enrolled patients who presented to our emergency department due to traumatic fractures between 2016 and 2017. We collected urine samples within 24 h after the patients had undergone surgery to determine the presence of lipiduria. Hypoxemia was defined as an SpO_2_ <95% determined with a pulse oximeter during the hospitalization. Patients’ anthropometric data, medical history, and laboratory test results were collected from the electronic medical record. Logistic regression analyses were used to determine the associations of clinical factors with postoperative lipiduria and hypoxemia with multivariate adjustments. A total of 144 patients were analyzed (mean age 51.3 ± 22.9 years, male 50.7%). Diabetes (odd ratio 3.684, 95% CI, 1.256–10.810, *p* = 0.018) and operation time (odd ratio 1.005, 95% CI, 1.000–1.009, *p* = 0.029) were independently associated with postoperative lipiduria, while age (odd ratio 1.034, 95% CI, 1.003–1.066, *p* = 0.029), body mass index (odd ratio 1.100, 95% CI, 1.007–1.203, *p* = 0.035), and operation time (odd ratio 1.005, 95% CI, 1.000–1.010, *p* = 0.033) were independently associated with postoperative hypoxemia. We identified several factors independently associated with postoperative lipiduria and hypoxemia in patients with fracture undergoing surgical intervention. Operation time was associated with both postoperative lipiduria and hypoxemia, and we recommend that patients with prolonged operation for fractures should be carefully monitored for clinical signs related to fat embolism syndrome.

## Introduction

Fat embolism syndrome (FES) is a clinical condition that arises as a consequence of fat globules in the systemic circulation (1, 2). It usually developed after orthopedic trauma, such as long bone fractures (3, 4). The clinical presentations of FES may vary widely, while the diagnostic criteria remain ill-defined (5). This may result in a wide range of reported incidences of this serious complication (4). The incidence of FES was found to be less than 1% in patients with orthopedic fractures (4, 5). Nevertheless, the rate could be as high as 30% in patients with multiple fractures (6, 7). Moreover, FES is associated with a high mortality rate (8), though its treatment is largely supportive.

The proposed pathophysiology of FES (5) includes endothelial injury followed by release of inflammatory cytokines and acute respiratory distress, hypoxemia, neurological deficit, thrombocytopenia and disseminated intravascular coagulation, all of which may contribute to detrimental outcomes. Currently, there is no established treatment for FES to improve its outcomes, and its care mainly involves supportive measures (5, 9). Early awareness of patients who are at risk for FES might be helpful. Unfortunately, the pathophysiologic mechanisms of FES have not been well-established (5).

Among the proposed diagnostic criteria of FES (5, 10), respiratory involvement (hypoxemia) is an important manifestation, which may result from endothelial injury and subsequent acute respiratory distress syndrome (5). Lipiduria (5) is another criterion for the diagnosis of FES that may be related to renal endothelial injury. Both lipiduria and hypoxemia are not well studied in patients with orthopedic fractures. As postoperative hypoxemia is not uncommon (11), we investigated factors associated with postoperative lipiduria and hypoxemia in patients undergoing surgery for orthopedic fractures in this study.

## Materials and Methods

This study was conducted in accordance with the Declaration of Helsinki. The study protocol was approved by the Institutional Review Board of Taichung Veterans General Hospital, Taichung, Taiwan (approval number: SF17001B). All patients provided written informed consent. We enrolled patients who presented to our emergency department due to traumatic fractures between 2016 and 2017. After initial assessment and preoperative preparation, these patients underwent surgical intervention for their fractures and were admitted. Patients’ anthropometric data, medical history, and laboratory test results were collected from electronic medical records.

**Figure 1:**
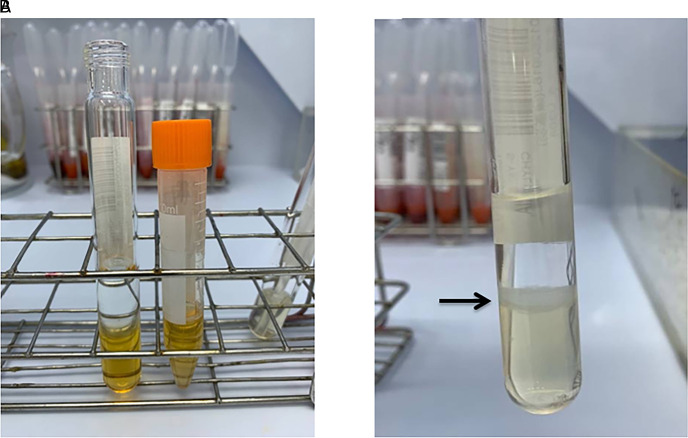
(**A**) A urine sample before the experiment and (**B**) after mixing with 5mL diethyl ether (E Merck, D-6100 Darmstadt, F.R. Germany) and centrifugation at 1,500rpm for 2min. The black arrow indicates the opaque layer we extracted for determination of lipiduria.

**Figure 2:**
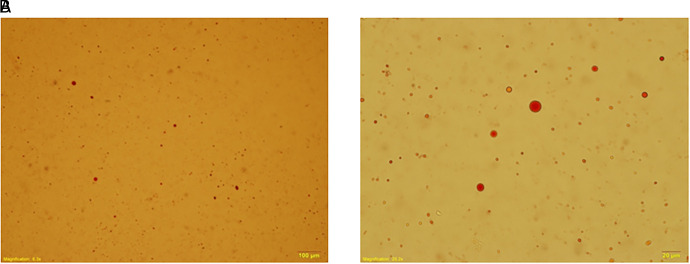
A urine sample stained with neutral fat stain solution observed using a microscope. (**A**) 100×. (**B**) 400×.

We collected urine samples within 24 h after the operation to determine the presence of lipiduria. Approximately 5 mL of urine sample was mixed with 5 mL diethyl ether (E Merck, D-6100 Darmstadt, F.R. Germany), and the mixture was centrifuged at 1,500 rpm for 2 min (**Figure 1**). We removed the upper layer of transparent fluid, and extracted the opaque layer for determination of lipiduria. Neutral fat stain solution was added to the samples, which were then examined using a microscope. **Figure 2** shows a sample with positive neutral fat stain.

SpO_2_ <90% (∼PaO_2_ <60 mm Hg) (12) has been used to define FES (13). In this study, we defined hypoxemia as an SpO_2_ <95% (14) determined with a pulse oximeter during the hospitalization. The monitoring of SpO_2_ was accompanied with vital signs measurements (usually every 6–8 h). More frequent monitoring may be required, depending on the patients’ clinical condition. Patients’ renal function was determined using estimated glomerular filtration rate (eGFR) according to the Chronic Kidney Disease Epidemiology Collaboration (CKD-EPI) equation (15). Information about operation time, blood loss, and blood transfusion was recorded according to the operation notes.

The statistical analyses were conducted using the Statistical Package for the Social Sciences (IBM SPSS version 22.0; International Business Machines Corp, NY, USA). We divided our patients into two groups according to whether or not they had lipiduria within 24 h after the operation. The statistical differences in continuous and categorical variables between the two groups were examined using the Student's t test and the Chi-square test, respectively. Logistic regression analyses were used to determine the associations of clinical factors with postoperative lipiduria and hypoxemia with multivariate adjustments. In all of the analyses, a two-sided P value of less than 0.05 was considered statistically significant.

## Results

A total of 144 patients were analyzed (mean age 51.3 ± 22.9 years, male 50.7%). **Table 1** shows the characteristics of the study population according to whether or not they had lipiduria after the surgery. Patients who had lipiduria after operation had a higher proportion of diabetes (25.0% vs. 11.3%, *p* = 0.030), a longer operation time (214 ± 124 vs. 174 ± 84 min, *p* = 0.027), and a higher rate of postoperative hypoxemia (SpO_2_ <95%) (40.6% vs. 20.0%, *p* = 0.007) compared with those who had no lipiduria. There were no significant between-group differences in the other variables.

**Table 1:** Characteristics of the study population according to postoperative lipiduria.

Variables	Lipiduria (−)	Lipiduria (+)	*P* value
*N*	80	64	
Age, years	51.3 ± 23.0	51.2 ± 22.8	0.974
Male sex, *n* (%)	36 (45.0)	37 (57.8)	0.126
Body mass index, kg/m^2^	24.1 ± 4.4	25.6 ± 5.1	0.055
Smoking, *n* (%)	8 (10.0)	5 (7.8)	0.649
Diabetes, *n* (%)	9 (11.3)	16 (25.0)	0.030
Hypertension, *n* (%)	19 (23.8)	15 (23.4)	0.965
White blood cell, per µL	11,525 ± 4,572	12,246 ± 5,473	0.390
Hemoglobin, g/dL	13.1 ± 1.9	13.2 ± 2.1	0.762
Platelet, 10^3^/µL	221 ± 64	241 ± 79	0.096
eGFR, mL/min/1.73 m^2^	92.8 ± 28.1	93.5 ± 24.7	0.869
ALT, U/L	28.9 ± 27.9	40.0 ± 47.0	0.087
Blood glucose before operation, mg/dL	130 ± 34	134 ± 44	0.642
Long bone fracture, *n* (%)	67 (83.8)	56 (87.5)	0.526
Blood loss during operation, mL	220 ± 284	279 ± 357	0.279
Blood transfusion, *n* (%)	18 (22.5)	12 (18.8)	0.615
Operation time, min	174 ± 81	214 ± 124	0.027
SpO_2_ <95% after operation, *n* (%)	16 (20.0)	26 (40.6)	0.007

*Values are mean ± SD or n (%). ALT, alanine aminotransferase. eGFR, estimated glomerular filtration rate.*

**Table 2** shows the associations between clinical factors and postoperative lipiduria. In the univariate analysis, diabetes (odd ratio 2.630, 95% CI, 1.074–6.436, *p* = 0.034) and operation time (odd ratio 1.001, 95% CI, 1.000–1.007, *p* = 0.025) were significantly associated with postoperative lipiduria. The associations of diabetes (odd ratio 3.684, 95% CI, 1.256–10.810, *p* = 0.018) and operation time (odd ratio 1.005, 95% CI, 1.000–1.009, *p* = 0.029) with postoperative lipiduria remained significant after adjustments for age, sex, and other relevant variables (**Table 2**).

**Table 2:** Associations between clinical factors and postoperative lipiduria (dependent variable).

Independent variables	Univariate analysis		Multivariate analysis^a^	
	Odds Ratio (95% CI)	*P*	Odds Ratio (95% CI)	*p*
Age (year)	1.000 (0.985–1.014)	0.974	1.012 (0.986–1.039)	0.361
Sex (male vs. female)	1.675 (0.863–3.252)	0.128	1.971 (0.863–4.503)	0.107
Body mass index (kg/m^2^)	1.072 (0.997–1.152)	0.059	1.069 (0.989–1.154)	0.092
Smoking (yes vs. no)	0.763 (0.237–2.455)	0.650	0.319 (0.073–1.385)	0.127
Diabetes (yes vs. no)	2.630 (1.074–6.436)	0.034	3.684 (1.256–10.810)	0.018
Hypertension (yes vs. no)	0.983 (0.453–2.132)	0.965	0.801 (0.293–2.189)	0.666
eGFR (mL/min/1.73 m^2^)	1.001 (0.989–1.014)	0.868	1.004 (0.983–1.027)	0.687
Operation time (min)	1.004 (1.000–1.007)	0.025	1.005 (1.000–1.009)	0.029

*eGFR, estimated glomerular filtration rate.*

*^a^* *Adjusted for age, sex, body mass index, smoking, diabetes, hypertension, eGFR, and operation time.*

We examined the associations between clinical factors and postoperative hypoxemia (SpO_2_ <95%), as shown in **Table 3**. Significant associations of age, diabetes, hypertension, eGFR, and operation time with postoperative hypoxemia were noted in the univariate analysis. After adjustments for relevant variables, age (odd ratio 1.034, 95% CI, 1.003–1.066, *p* = 0.029), body mass index (odd ratio 1.100, 95% CI, 1.007–1.203, *p* = 0.035), and operation time (odd ratio 1.005, 95% CI, 1.000–1.010, *p* = 0.033) were independently associated with postoperative hypoxemia.

**Table 3:** Associations between clinical factors and SpO_2_ <95% after operation (dependent variable).

Independent variables	Univariate analysis		Multivariate analysis^a^	
	Odds Ratio (95% CI)	*P*	Odds Ratio (95% CI)	*p*
Age (year)	1.027 (1.010–1.045)	0.002	1.034 (1.003–1.066)	0.029
Sex (male vs. female)	0.641 (0.311–1.323)	0.229	1.009 (0.402–2.535)	0.984
Body mass index (kg/m^2^)	1.061 (0.984–1.143)	0.122	1.100 (1.007–1.203)	0.035
Smoking (yes vs. no)	0.183 (0.023–1.454)	0.108	0.117 (0.012–1.160)	0.067
Diabetes (yes vs. no)	2.738 (1.128–6.649)	0.026	1.417 (0.504–3.985)	0.509
Hypertension (yes vs. no)	2.872 (1.285–6.419)	0.010	1.270 (0.459–3.516)	0.645
eGFR (mL/min/1.73 m^2^)	0.980 (0.966–0.994)	0.006	0.992 (0.970–1.015)	0.512
Operation time (min)	1.005 (1.000–1.009)	0.030	1.005 (1.000–1.010)	0.033

*eGFR, estimated glomerular filtration rate.*

*^a^* *Adjusted for age, sex, body mass index, smoking, diabetes, hypertension, eGFR, and operation time.*

## Discussion

In this study, we demonstrated that diabetes and operation time were associated with postoperative lipiduria in patients with fractures undergoing surgical intervention (**Table 2**). Moreover, operation time was independently associated with postoperative hypoxemia (**Table 3**). Both postoperative lipiduria and hypoxemia are common presentations of FES (5, 11), which is an uncommon but severe postoperative complication with a poor prognosis (8, 16). Our findings are clinically relevant and might help identify patients with high risk of postoperative FES.

Lipiduria has been associated with nephrotic syndrome (17). The cause of lipiduria in nephrotic syndrome may be secondary to hyperlipidemia (18). Our finding that diabetes was associated with lipiduria (odd ratio 3.684, 95% CI, 1.256–10.810, *p* = 0.018, **Table 2**) is perhaps not surprising, as diabetes has been associated with proteinuria and nephrotic syndrome (19). We speculate that an increase in oxidative stress after acute trauma and surgical intervention (20–22) might be related to postoperative lipiduria. Oxidative stress has been reported to contribute to acute kidney injury after orthopedic trauma in an animal model (23). Oxidative stress may result in an increase in glomerular permeability (24), which has been associated with lipiduria (25, 26). This scenario may help explain the finding in a recent study (16) that showed delayed time to operation was associated with FES in patients with acute trauma, and may also account for our result, which indicated operation time (odd ratio 1.005, 95% CI, 1.000–1.009, *p* = 0.029) was independently associated with postoperative lipiduria.

Moreover, we found that operation time was independently associated with postoperative hypoxemia (odd ratio 1.005, 95% CI, 1.000–1.010, *p* = 0.033). An increase in oxidative stress associated with surgical intervention could lead to an increase in pulmonary permeability (22), which in turn may result in postoperative hypoxemia. Both age and body mass index were also independently associated with postoperative hypoxemia, and these findings were consistent with previous reports (27–29). Twenty-five of our patients had both lipiduria and hypoxemia after surgery for their fractures. The mean operation time of these patients was 227 ± 130 min, compared with 178 ± 82 min (*p* = 0.034) for the 64 patients who did not have postoperative lipiduria and hypoxemia. As bone marrow manipulation during orthopedic surgery might increase the risk of FES (30), it is reasonable to postulate that this could explain the association of operation time with postoperative lipiduria and hypoxemia. Based on our findings, we recommend that patients who had prolonged operation for fractures should be carefully monitored for clinical signs related to the FES.

There were several limitations in this study. First, the number of our study patients was relatively small. As the incidence of FES was less than 1% (4, 5), a larger number of patients are needed to investigate predisposing factors of this serious complication. Second, we did not investigate proteinuria and lipiduria before the operation for all study patients. We cannot exclude the possibility that some of our patients had preoperative lipiduria which was not related to the operation. Third, we used SpO_2_ <95% determined using a pulse oximeter, rather than low PaO_2_ determined by an arterial blood gas analysis, as the definition of hypoxemia. This must be acknowledged as a potential confounder, although low SpO_2_ (<90%) was used to define FES in a recent study (13).

## Conclusion

In summary, we identified several factors independently associated with postoperative lipiduria and hypoxemia in patients with fracture undergoing surgical intervention. Operation time was associated with both postoperative lipiduria and hypoxemia, and we recommend that patients with prolonged operation for fractures should be carefully monitored for clinical signs related to FES.

## Data Availability

The datasets presented in this article are not readily available because of privacy restrictions. Requests to access the datasets should be directed to the corresponding author.
